# Machine learning prediction of academic collaboration networks

**DOI:** 10.1038/s41598-022-26531-1

**Published:** 2022-12-20

**Authors:** Giuliano Resce, Antonio Zinilli, Giovanni Cerulli

**Affiliations:** 1 Department of Economics, University of Molise, Campobasso, Italy; 2 Research Institute on Sustainable Economic Growth, National Research Council of Italy, Rome, Italy

**Keywords:** Complex networks, Statistics

## Abstract

We investigate the different roles played by nodes’ network and non-network attributes in explaining the formation of European university collaborations from 2011 to 2016, in three European Research Council (ERC) domains: Social Sciences and Humanities (SSH), Physical and Engineering Sciences (PE), Life Sciences (LS), as well as multidisciplinary collaborations. On link formation in collaboration networks, existing research has not yet compared and simultaneously examined both network and non-network attributes. Using four machine learning predictive algorithms (LASSO, Neural Network, Gradient Boosting, and Random Forest) our results show that, over various model specifications: (i) best model link formation accuracy is larger than 80%, (ii) among the non-network attributes, public funding plays an important role in PE and LS, (iii) network attributes count more than non-network attributes for the formation, sensibly increasing accuracy, (iv) feature-importance scores show a different ordering in the four domains, thus signalling different modes of knowledge production and transmission taking place within these different scientific communities.

## Introduction

Studies on network dynamics have gained increasing relevance in various scientific domains. In physics, engineering, biology, as well as in the social sciences, in fact, understanding networks’ structure, evolution, and endogenous dynamics have become a central issue. From a methodological perspective, there are different ways to investigate network dynamics. Link formation (or link prediction), which studies the configuration of current and future connections between agents, is currently one of the most promising research areas in knowledge and innovation networks^1–5^.

In these footsteps, this paper contributes to the literature on network formation focusing on a specific network type, i.e. the “knowledge” networks. It investigates the relative importance of some network topological measures (endogenous) and exogenous attributes in explaining the formation of collaborations in a large set of European universities in different scientific domains and collaboration environments. As far as we are aware, research has only really looked at one of the two mechanisms (endogenous or exogenous). Comparing simultaneously the significance of these two mechanisms in link formation is one of the paper’s contributions. Because co-authorship and co-publication are both concrete and well-recognized forms of scientific collaboration, they are both used interchangeably in this paper^6^. By combining several sources coming from the EU-funded project $\hbox {RISIS}\dag$, we used the CWTS Publication database, which is a complete version of the Web of Science (WoS) devoted to bibliometric analyses, and the RISIS-ETER database, which contains data on higher education institutions in Europe. We build up a dataset which includes in-formation on collaboration networks of 1443 European universities over the period 2011–2016. We differentiate our analysis by considering co-publications falling within the three European Research Council (ERC) scientific domains (or ERC classification): Social Sciences and Humanities (SSH), Physical and Engineering Sciences (PE), and Life Sciences (LS), as well as papers stemming from multidisciplinary journals (MS). These four networks pursue specific and different modes and practices of knowledge production and exchange, as diverse is their logic of activating collaborations. Studying collaborations through the lens of common scientific publications is relevant especially from a public funding perspective, given the policymakers’ concern about facilitating collaborations and supporting the formation of sustainable network links (Davies et al. 2022). Furthermore, studying collaboration network is extremely crucial for technology development and deployment^7^.

By exploiting the knowledge of past networks’ configurations, link formation aims at shedding light on two related aspects: on the one hand, what are the most relevant drivers of link creation (data-driven *preferential attachment*), either endogenous (network topological features), and/or exogenous (nodes’ external-to-network characteristics); on the other hand, for every single node, what are the attachment probabilities to all the other nodes of the network, either for incumbent nodes—nodes that are already part of the extant network—and/or for newcomer nodes—nodes that are not part of the current network yet, but that are likely to become part of.

Reaping the benefits of organizational collaborative networks requires the research network to remain open and easily accessible to newcomers. For this reason, by means of recent developments in machine learning predictive algorithms, this paper attempts to estimate the probability that a university, let’s say “*x*”, collaborates with a university, let’s say “*y*”, by considering the past centrality and the sharing of common neighbors (notice that newcomers have not this information), as well as their idiosyncratic attributes (with newcomers having this information). By disentangling the contribution of endogenous and exogenous attributes, we are able to measure to what extent link prediction is accurate for the incumbent as well as for newcomers.

To this aim, we develop three different models: a full model, a model with only endogenous variables, and a model with only exogenous variables. Disentangling the effect of endogenous and exogenous variables we are able to isolate the contribution of network properties and external attributes in the link formation. On these models, we compare the prediction performances of four machine learning predictive algorithms (LASSO, Neural Network, Gradient Boosting, and Random Forest) in order to validate the results and in order to highlight the different performances each algorithm can have on different models and different ERC scientific domains.

The remainder of the paper is structured as follows. The next section illustrates the literature review. “Data and methods” describes definitions and notations, the data, and methods. “Results and discussion” presents the main results, and concluding thoughts are offered in “Discussion”.

## Conceptual framework

Link creation has been investigated using various network formation processes. A large and growing body of empirical research highlights how the similarity notion, operationally based on nodes’ similar characteristics, can influence the formation of relationships^8–10^. A common mechanism, often used to explain how links are formed in scientific networks, is the so-called *preferential attachment*^11,12^. Also, collaboration may be activated on the basis of common neighbours or similar exogenous features^9,13^. Other mechanisms emphasize the role of the past network topological structures, such as cyclical forms, and hierarchical structures^14,15^, whereas other authors have emphasized the role of node’s centrality as a measure affecting the connection process^16–18^. For a review of the different mechanisms of network link formation and prediction, one can refer to^2^, and^19^.

In the last years, international organizations (e.g. European Commission, Research Funding Organizations) that deal with science, technology, and innovation have recognized the significance of scientific collaboration because it is seen as a major driver of innovation and a crucial element in the advancement of technology^7^. These reasons are why the European Research Area has made fostering scientific cooperation one of its main objectives^20^. The growth in national and international context of funding instruments designated exclusively for the collaboration promotion between organizations serves as a sign of this better awareness^21,22^. Collaborations among universities and other public and private organizations are at the center of the policymakers’ agenda, especially with the aim of fostering the integration of research activities among European organizations (EUROPEAN COMMISSION, 2020. A New Era for Research and Innovation. Com2020). Furthermore, the changes that took place in recent years within the scientific ecosystem have led universities to act more and more strategically^23,24^, by planning strategies to foster participation and collaborative research with the explicit aim of improving both research quality and quantity. For all of these reasons, it is necessary to study the factors that affect the interchange of knowledge, the generation of new ideas, the promotion of innovation, and the generation of economic benefit^25^.

Collaboration among universities is an important lever of knowledge production and diffusion, as each academic organization is a network’s node linked to other nodes via joint publications. The number of publications is a measure of success for every researcher and for the university the researcher belongs to. A range of previous works has argued that papers with more authors tend to be cited more frequently than papers with only one author^26^, and have a higher research impact in terms of the number of publications^6,27^. Other scholars have studied co-publication network for exploring what factors affect the motivation to engage into scientific collaborations^28,29^. Some of these studies have found evidence of the existence of the so-called *Matthew effect* in co-authorship networks^30,31^, that is, patterns of self-reinforcing inequality related, in this case, to knowledge acquisition. When this process takes place in combination with a competitive environment of scientific publishing (the so-called “publish or perish” phenomenon), the Matthew effect can produce even further inequalities, engendering publishing polarized performance.

This paper wants to stress the contribution of network attributes in explaining collaboration among European universities in collaboration networks. We look at the link prediction problem with the double lens of the social network theory^13,32^ and organizational studies in the knowledge context. Given the shortage of papers on link formation at the university level, we focus on those factors (both endogenous and exogenous) identified by the literature as prominent in explaining the probability for a university to form a link. Among the exogenous ones, an important feature is the size of a university, as larger universities tend to attract more collaborations^33,34^. Similarly, universities’ scientific reputation is another important driver of collaboration formation^33,35^. Some papers studied the bias towards collaborating with research partners (at researcher and organizational level) with similar reputation generating cumulative advantages^25,36^. Other studies focused on the geographical proximity between organizations, as closer organizations tend to be more prone to collaborate^37,38^. Only a few papers have looked at the correlation between funding and scientific collaboration^39,40^, but it has recently gained significant attention in scientific politics. These papers focused mainly on STEM fields and are single-country studies. The relationship between funding and collaboration is still not fully understood and has not been thoroughly investigated, particularly in its international dimension. This study aims to fill this gap in the literature: controlling for size, university reputation, GDP per capita and geographical proximity, we test how the share of public funding affects the co-publication among universities in four scientific networks. We therefore start by asking:

1. does the share of public funding received by the university matter when creating co-publication links between universities?
2. does public funding have a different effect on the considered scientific fields?

The questions are responded to by analysing the government funding received by each university. According to Adams et al. (2005), top universities academic departments receiving more government funds frequently take part in larger teams and tend to active new collaborations. Defazio et al. (2009) suggest that public funding may be a crucial component in creating more productive research network collaborations in Europe. Furthermore, Jeong et al. (2014) and Jeong and Choi (2015) investigated how spurious effects interact with funding to influence international research collaboration. The research on how funding influences collaboration is especially relevant. Constraints faced by organizations and researchers in the different European countries are typically characterized by heterogeneity. Public funding sources are not always as prevalent as they are in other countries. Additionally, the effects of regular funding can vary by scientific area (natural sciences vs social sciences, for instance)^41^. Based on this discussion, we empirically investigate whether the public funding at university level affects the link formation. So, we suggest that pure public funding has a positive and strong effect on the formation of links:

1. Link formation, particularly in PE and LS, is significantly affected by public funding.
2. In fields of science where long-term projects (such those in the life sciences or experimental physics) and massive research infrastructures are common, we expect that public funding is much more significant. Studies on R&D and innovation networks from the perspective of network science have revealed that actors are more likely to form a connection when they share common neighbors^42^. It is well known that scientific networks are organized into modules or clusters made up of nodes with a strong relation^43–45^. This evidence emerged from a literature which generally stresses the complex nature of collaboration in scientific contexts. Several studies identify the Jaccard coefficient, the betweenness centrality, and the cosine similarity as powerful endogenous factors affecting link prediction in scientific contexts^46^. For these reasons, we perform two measures of community detection on the four empirical networks: betweenness centrality in identifying researchers who participate in different communities and the Jaccard coefficient, also known as the community coefficient, which determines the proportion of shared researchers. Studies on knowledge networks also show that these networks are made up of a stable community of individuals who continue to publish together regularly^25,47^. Different collaboration practices have been revealed by other studies. A recent analysis of the lifetime careers of 3,860 computer scientists revealed that about the 73% of authors have worked with the same scientists just once and never again^48^. Different approaches to scientific collaboration, particularly between different scientific fields, have been observed^25,49^. This last aspect is addressed in this work by investigating historical connections using the lagged dependent variable. According to the cited literature, link prediction could be explained by different factors, either endogenous (e.g. past connection, centrality measures, common neighbours) or exogenous (node attributes). Both endogenous and exogenous characteristics, taken independently, are able to predict the topology of the observed knowledge network. There are no studies that we are aware of that combine endogenous and exogenous variables in analyses of co-authorship among European universities. To fill the aforementioned research gap, the following question arises:
3. which dimension-endogenous or exogenous-weighs more heavily in the prediction of the links? From the literature listed above, the neighborhood-based and community topological features in co-authorship networks are important latent features that can show strategic mechanisms for predicting collaborations much more than spatial proximity or field similarity features^50^. It is thus important to understand and disentangle the role of both endogenous and exogenous attributes in the link prediction process^19^. We expect the endogenous characteristics (previous co-authorship and network properties) to be highly conducive to better link prediction, more than the exogenous ones can do.
4. Endogenous features are more relevant to link prediction than exogenous factors are. In this way, we deem to contribute to the existing findings on collaboration networks by providing new insights on the role played by endogenous attributes in the four above mentioned scientific domains, thus shedding further light on the main drivers underlying the collaboration phenomenon. Our findings help policy makers design better research policy by revealing which aspects (both endogenous and exogenous) appear to encourage collaboration the most.

The analysis of the factors predicting network connections can shed light also on the issue of research *fragmentation*, thus conveying useful policy recommendations about how to facilitate knowledge diffusion, the combination of innovative ideas from different scientific communities, as well as participation of less connected entities. All these aspects are key to favor inclusiveness and integration, especially for newcomers^51,52^.

## Data and methods

### Definitions and notations

We define a network as a graph $G=(X,E)$, where *X* is a set of entities (nodes or vertices), and *E* indicates the link between these entities (edges or ties). In the context of our analysis, a node represents the university where a researcher (i.e., a author) works, whereas a link connects two universities if it exists at least one paper co-authored by researchers (co-authors) from two or more universities. Because we assume reciprocal collaboration, these networks are *undirected*. Moreover, our analysis does not consider loop and multiple connections among nodes. For this purpose, we attempt to achieve a high prediction accuracy in the formation of links for newcomers (e.g. actors that were not part of the observed network) at a reasonable computational cost.

By combining factors that measure different features of nodes and links into a single data frame, our objective is to predict the link formation between two nodes in four different collaboration graphs: Social Sciences and Humanities (SSH), Physical and Engineering Sciences (PE), Life Sciences (LS) and Multidisciplinary Science (MS).

Given a snapshot of the network at time *t*, we want to predict what links are likely to take place (or, possibly, to be kept) in a future time $t+1$. As mentioned in the previous section, many studies have tried to singling out what are the main features shaping knowledge networks structure. In this paper, two types of attributes are assumed to be crucial in driving link formation: endogenous attributes (previous co-authorship and previous network proprieties), and exogenous attributes (external-to-network features).

By means of some machine learning predicting algorithms, we are able to determine—for each node in each of the four networks—all the possible link probabilities with the other nodes, and provide an assessment of how much these probabilities are accurately estimated. This is the purpose of the first part of this work. Clearly, the accuracy thus obtained is conditional on the features used in the analysis (either endogenous or exogenous), and the characteristics of the scientific community (represented by the four domains).

Disentangling the role played by the endogenous and the exogenous attributes in all four ERC domains is key for our study, as we can isolate the contribution to link prediction stemming from the network proprieties and the external characteristics of the nodes. This distinction is at the basis of a fundamental predictive trade-off that we aim at exploring in this study: the knowledge of endogenous attributes is likely to increase prediction performance, but prevents to predict link formation for newcomers (i.e. nodes that did not belong in the observed networks); in contrast, the sole knowledge of exogenous (i.e., non-network) attributes allows to generate prediction also for newcomers, but at the cost of possibly reducing link prediction accuracy. Our study investigates this trade-off that has crucial policy relevance for steering future collaborative network configurations.

Finally, we provide an in-depth exploration of the contribution of each single predictor in driving the probability of setting-up a scientific collaboration. This makes it possible to emphasize the differences among the four ERC domains in terms of feature contribution. Results are interpreted and commented in the light of the current literature on knowledge networks.

### Data description

In order to collect the appropriate collaboration and organizational data, we use two large datasets coming from the EU-funded project RISIS: CWTS Publication and RISIS-ETER, which form together a unique tool of investigation for publication network at organizational level, covering the years 2011–2016. CWTS Publication collects a large body of Web of Science (WoS) information, while RISIS-ETER (database on European Higher Education Institutions) collects information about all European universities^53^. The main strength of this database is the fact that it provides harmonized data at Higher Education level for all European countries. We combined CWTS and RISIS-ETER using the same university ID.

To carry out our analyses, we performed a one-mode projection of the CWTS Publication dataset; in this way, links are established between universities participating to the same publication. In our analysis, the node is the university, and the link indicates a collaboration in a publication. Each research article was assigned to the specific ERC domain (SSH, PE, LS) through the categories made available by WoS (https://images.webofknowledge.com/images/help/WOS/hp_subject_category_terms_tasca.html). We also considered articles attributed to the multidisciplinary category (MS) according to the WoS classification.

Our dependent variable is a binary one, taking values 0 (no existence of a co-authorship), and 1 (existence of a co-authorship). To predict the link occurrence, for all four networks, we use the same model specification, made to the following features (or predictors):

- Exogenous features:
  - *gross domestic product* (PPS per inhabitant) of the region (NUTS 2) where the university is located (from ETER database);
  - *population density* of the region (NUTS 2) where the university is located (from ETER database);
  - *geographical proximity* between two universities in terms of kilometers (using coordinates from ETER database). We transformed this measure into its inverse;
  - *core funding*, indicating the overall government funding available for a university (from ETER database);
  - *citation score*, measured by the “mean normalized citation score”, which is the average number of citations of a university (from ETER database). We take this variable as a proxy of university reputation;
  - *number of students* by ERC domain (from ETER database), considered as a proxy of university size, properly scaled according to the three ERC domains (SSH, PE, LS). Notice that the MS domain has not been scaled.
- Endogenous features
  - *betweenness centrality*, defined as the proportion of the shortest paths between all couples of nodes that pass through a given actor in the network^54^;
  - *Jaccard coefficient*, indicating the similarity between the neighbourhood of two nodes. By “neighbourhood” we mean the adjacent vertices of a specific node;
  - *past connection* between two nodes, that identifies the memory of the link.

### Prediction task

Consider a pair of universities, let’s say *x* and *y*, observed at time *t*, that we indicate as $(x,y)_{t}$. To this pair, we associate a target binary variable $P_{(x,y)_{t}}$ (“making a join publication”) that takes values 1 (positive occurrence) if the pair has a co-publication, and value 0 (negative occurrence) otherwise. Based on the set of (lagged) features $({features_{(x,y)_{T<t}}})$ referring to the pair $(x,y)_{t}$, our prediction task is to find a mapping function *f*(.) (i.e., an machine learning binary classifier) that predicts as better as possible the co-publication event (i.e., the two-class variable $P_{(x,y)_{t}}$):

1 $$\begin{aligned} \begin{aligned} \big \{{features}_{(x,y)_{T<t}}\big \}\xrightarrow {f(.)} P_{(x,y)_{t}}. \end{aligned} \end{aligned}$$

The features used to predict $P_{(x,y)_{t}}$ are those listed in the previous section and refer to a set of both exogenous and endogenous (network) features. We organize the database in the edge list format: the features are by column, and the couple of nodes are by row. In the predictions, three features (*geographical proximity*, *Jaccard coefficient*, and *past connection*) are identified uniquely for each couple of nodes, while the other features enter into the model twice as they are defined for both the nodes (universities).

The standard ML procedure to first train, and then test the mapping function is that of randomly splitting the data in a *training set*, over which the model is estimated and tuned, and a *testing set*, over which its predictive power is tested^55^. The size of these two sets must be chosen taking into account the trade-off between the benefit of a large training set (i.e., more information to build the mapping in (1)), and the benefit of a sufficiently large testing set (i.e., more information for a more precise estimation of the testing error). To account for this trade-off, we follow the usual compromise of randomly dividing the database into a 70 percent of observations for training, and the remaining 30 percent as out-of-sample test set^56^.

To carry out our analysis, we use four different ML predicting algorithms for co-authorship prediction:

- Least Absolute Shrinkage and Selection Operator (LASSO): a regression statistical method that performs features selection and regularization with L1 norm to reduce over-fitting and increase prediction accuracy and interpretability^57^;
- Random Forest (RF): a family of randomized tree-based classifier decision trees which uses different random subsets of the features at each split in the tree^58^;
- Gradient Boosting Machines (GBM): an ensemble method which works in an iterative way where at each stage new learner tries to correct the pseudo-residual of its predecessors^59^;
- Neural Network (NN): a model that uses a set of connected input/output units in which each connection has an associated weight, and learns by adjusting the weights to predict the correct class label of the given inputs^60^.

The hyper-parameters’ optimization is carried out over the training set using a 10-fold cross-validation with 5 petitions. All models have been implemented using the R software, trained with the optimisation algorithms available through the $\texttt {caret}$ package^61^.

We also deal with target variable imbalance. Indeed, co-publications are largely fewer than non co-publications and this generally produces under-performing predictions, as the unconditional probability to co-publish is highly skewed towards the absence of co-publication, thus giving this category larger advantage when classifying new instances. To address the imbalance issue, all the four ML models have been estimated using Random Over-Sampling Examples (ROSE), an algorithm where artificial balanced samples are generated according to a smoothed bootstrap approach^62^.

The performance of co-publications’ classification prediction is assessed through two main performance measures: the Receiver Operating Characteristics (ROC) curve^63^, and the Precision-Recall (PR) curve^64^. The ROC curve shows the classifier diagnostic ability by plotting the true positive rate (TPR) on the *y*-axis against the false positive rate (FPR) on the *x*-axis, since its discrimination threshold is varied^65^. Although the ROC analysis has firm statistical interpretation, its results can be misleading when applied to an imbalanced classification setting^66^. A better alternative in this case is the PR curve^64^. The recall is the FPR, while the precision is defined as the ratio of the number of true positives and the total number of predicted positive samples. In the unpredictable case, the PR curve has a constant value equal to the ratio between the number of positive samples and the total number of samples.

Our machine learning models give information also on how useful each feature is in explaining co-publications prediction. Each model has a different algorithm to estimate the so-called feature-importance indices^55^. In LASSO, feature importance is estimated as the absolute value of the coefficients corresponding to the tuned model. In RF, feature importance is computed as the mean gain produced by the feature over all the trees, where the gain is measured by the Gini index. The feature importance in GBM is computed as the average improvement of the splitting on the features across all the trees generated by the boosting algorithm. The feature importance in NN, finally, is determined by identifying all weighted connections among the layers of the network.

## Results and discussion

This section presents our results on predicting collaboration links. Our focus will be on two main points: (i) the predictability of the links, and the computation of feature importance indices for the variables used for prediction purposes. We consider separately the four abovementioned scientific areas: Social Sciences and Humanities (“The predictability of the links and the feature-importance in social sciences and humanities (SSH)”), Physical and Engineering Sciences (“The predictability of the links and the feature-importance in physical and engineering sciences (PE)”), Life Sciences (“The predictability of the links and the feature-importance in life sciences (LS)”) and Multidisciplinary Sciences (“The predictability of the links and the feature-importance in multidisciplinary science”).

Table 1 sets out some co-authorship network descriptive statistics, specifically: the number of nodes, the number of links, and the network density. All the metrics in Table 1 represent the network size by year and domain. Network density describes the portion of the potential connections in a network with a specific number of nodes that are actual connections. It can be observed that the density increases over the years in all four scientific domains, thus indicating a reinforced interaction among the nodes. In a nutshell, we can see that the co-authorship network has become more integrated over time. In knowledge networks, a higher density can indicate an easier access to new knowledge and a higher efficiency of knowledge flows.

**Table 1:** Descriptive statistics for the European universities’ co-publication network.

Year	Nodes	Links	Density	Nodes	Links	Density
	SSH			PE		
2011	1322	11,946	0.0137	1320	38,637	0.0444
2012	1322	13,950	0.0160	1320	41,469	0.0476
2013	1322	15,604	0.0179	1320	44,754	0.0514
2014	1322	16,953	0.0194	1320	46,790	0.0537
2015	1322	18,194	0.0208	1320	48,892	0.0562
2016	1322	19,877	0.0228	1320	55,887	0.0642
	LS			MS		
2011	1250	35,481	0.0455	1223	24,329	0.0326
2012	1250	38,709	0.0496	1223	26,633	0.0356
2013	1250	41,509	0.0532	1223	28,373	0.0380
2014	1250	47,163	0.0604	1223	31,164	0.0417
2015	1250	50,901	0.0652	1223	39,324	0.0526
2016	1250	56,170	0.0720	1223	38,298	0.0513

Data by year and scientific domain.

Table 2 shows the number of new nodes and the proportion of edges that appeared between *t* and $t+1$. At each time step, new nodes and edges are added to the network. Based on the table, the number of new nodes does not increase with time, but the number of links from new nodes increases on average across all scientific domains.

**Table 2:** Number of new nodes and percentage of edges from new nodes over time and by scientific domain.

	**SSH**				
	2011->2012	2012->2013	2013->2014	2014->2015	2015->2016
New nodes	114	110	108	118	101
% new links from new nodes	2.8	2.7	3.4	4	3.3
	**PE**				
	2011->2012	2012->2013	2013->2014	2014->2015	2015->2016
New nodes	112	102	85	74	97
% new links from new nodes	1.9	2	1.9	2	2.7
	**LS**				
	2011->2012	2012->2013	2013->2014	2014->2015	2015->2016
New nodes	81	76	89	80	72
% new links from new nodes	1.6	1.9	2.1	2.7	2.5
	**MS**				
	2011->2012	2012->2013	2013->2014	2014->2015	2015->2016
New nodes	102	112	110	103	102
% new links from new nodes	2.7	4.6	4.6	4.3	5.3

New edges can emerge from and connect to any node, old or new.

We perform link prediction over three models (with all, with only the exogenous, and with only the endogenous features), and with the four ML algorithms presented in the previous section (that is, GBM, LASSO, NN, and RF), trained on 70% of observations and tested on the remaining 30%. The models are tuned via cross-validation, which trains and tests the model by tuning the hyper-parameters with the aim of maximising the area under the ROC curve.

### The predictability of the links and the feature-importance in social sciences and humanities (SSH)

**Figure 1:**
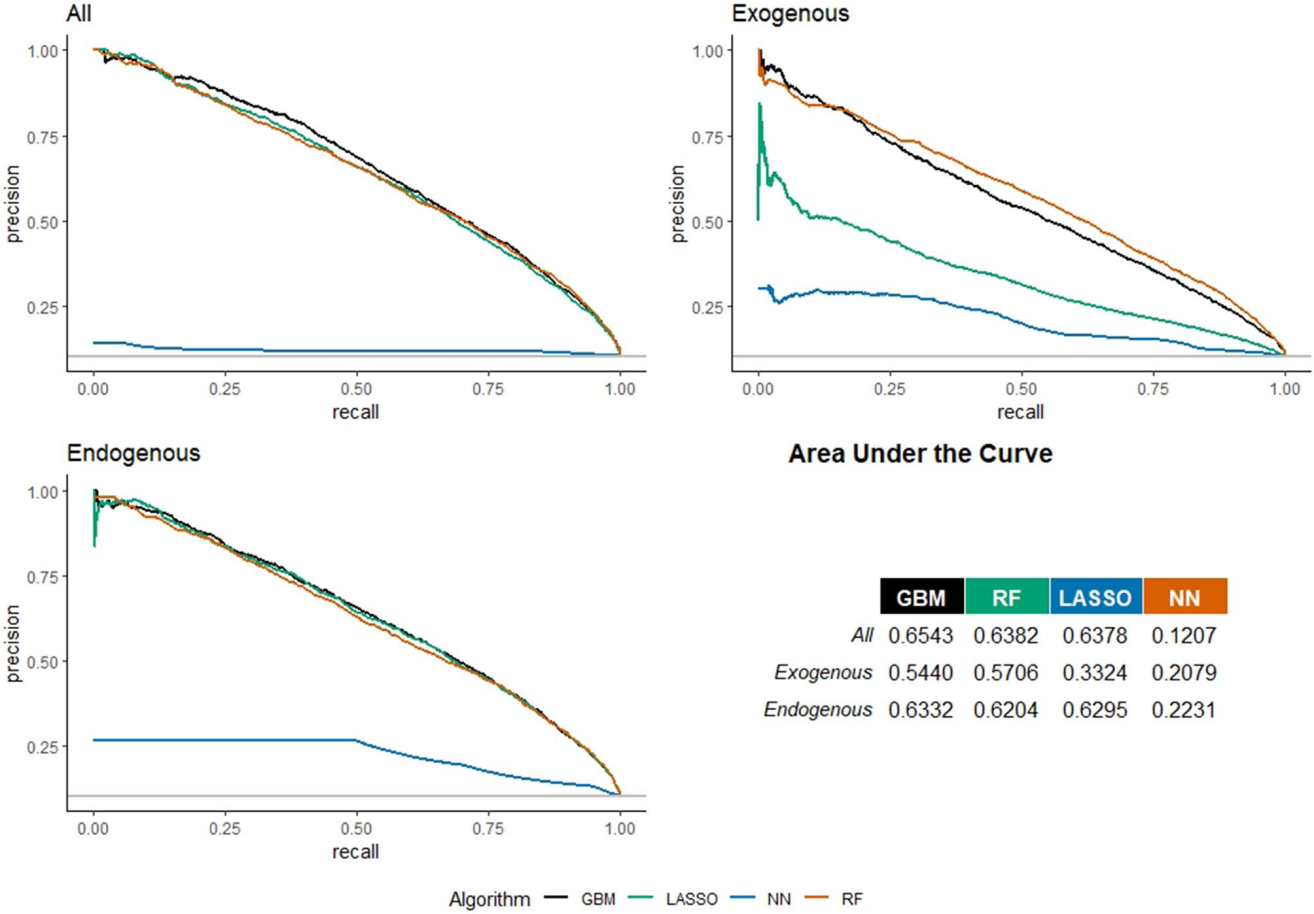
Precision–recall curves for European universities’ co-publication prediction in the Social Sciences and Humanities domain. Three models considered: with all, with only exogenous, and with only endogenous features.

Figure 1 shows the Precision-Recall curves for link prediction in the SSH area. For the model including all the features, the best algorithm in terms of Area Under the Curve (AUC) is the GBM (with a score equal to 0.6543), while considering only exogenous features, the best model in terms of AUC is the RF (0.5706); finally, considering only endogenous features, the best model is the GBM, showing an AUC of 0.6332. Using two existing link prediction algorithms with all features, the AUCs are lower than the AUC of the best ML algorithm (GBM): AUC for Logistic regression = 0.6409; AUC for Naive Bayes regression = 0.5890. There is a substantial worsening in the AUC when the endogenous features are removed (i.e., a drop of around 13%), while by excluding the exogenous features, thus retaining only network information, the AUC becomes closer to the AUC of the model with all features, with a smaller worsening (3%).

Considering the best predicting model, Table 3 lists a battery of accuracy performance indicators typically used in the machine learning literature. The area under the ROC curve and all the measures listed in Table 3 confirm that the model with all features outperforms the model with only endogenous features which, in turn, outperforms the model with only exogenous features. These results provide evidence on the relevance of the network structure for link prediction, vis-à-vis a model including only exogenous (out-of-network) information.

For all the models (with all, only exogenous, and only endogenous features), the accuracy is not larger than the no-information rate. This is a rather striking finding, that however may be explained by two aspects: the first regards the large prevalence of zeros in our target variable, making the no-information rate too high, with a value close to 0.9 indicating that only the 10% of observations owns a link; the second aspect is associated to the nature of the SSH sector, a scientific area where publications are largely underestimated by the WoS archive compared, in particular, to hard-science sectors. It is well recognized, in fact, that in the SSH academics prefer other types of scientific outputs (e.g. books, conference proceedings, gray materials) that the WoS is unable to capture.

**Table 3:** Accuracy performance indicators for European universities’ co-publication prediction in Social Sciences and Humanities domain.

Model	All	Exogenous	Endogenous
Best algorithm	GBM	RF	GBM
ROC	0.9153	0.8945	0.9111
Accuracy	0.8331	0.8102	0.8288
95% CI	(0.8291, 0.837)	(0.806, 0.8144)	(0.8248, 0.8328)
No information rate	0.8962	0.8947	0.8973
P-value [Acc > NIR]	1.0000	1.0000	1.0000
Sensitivity	0.8324	0.8091	0.8278
Specificity	0.8390	0.8196	0.8378
Pos pred value	0.9781	0.9744	0.9781
Neg pred value	0.3671	0.3356	0.3575
Prevalence	0.8962	0.8947	0.8973
Detection rate	0.7460	0.7240	0.7428
Detection prevalence	0.7627	0.7429	0.7595
Balanced accuracy	0.8357	0.8144	0.8328

Three models considered: with all, with only exogenous, and with only endogenous features.

**Figure 2:**
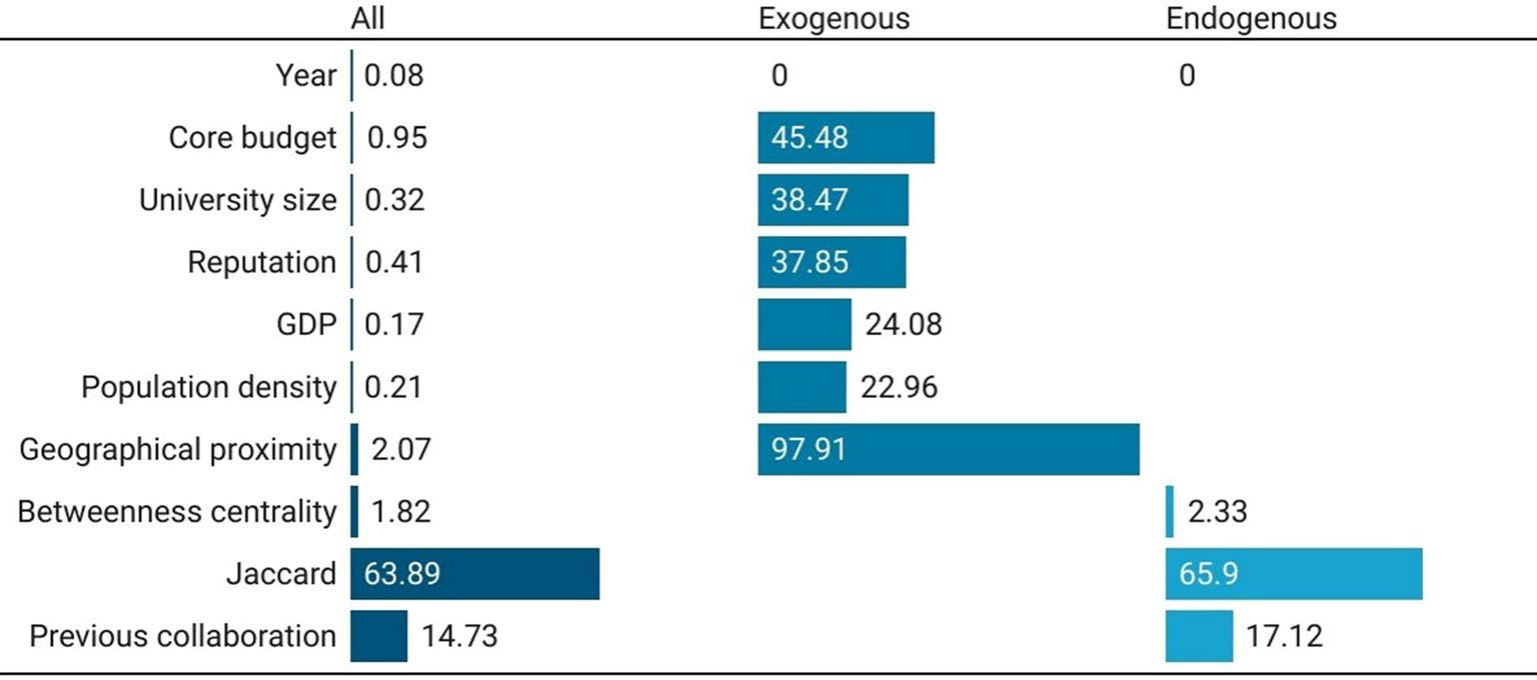
Feature-importance scores for European universities’ co-publication prediction in Social Sciences and Humanities. Three models considered: with all, with only exogenous, and with only endogenous features.

Figure 2 illustrates feature-importance scores for the best algorithm used in the three models (again, with all, only exogenous, and only endogenous features). The model with all features shows that the Jaccard coefficient is the most important feature, with a score of 63.89, followed by previous collaboration (score equal to 14.73) and geographical proximity (with a score of 2.07). The network structure thus seems to matter more than all the other features for co-authorship prediction. Excluding the geographical proximity, core budget, university size and reputation become the most important features to predict connections. As expected, the Jaccard index becomes the most relevant feature again when exogenous features are not included in the model. Universities with common neigbours are likelier to share the same scientific cluster. Pairs of universities with a higher Jaccard coefficient have a greater cognitive proximity, which reduces the diversification in favor of common goals. Having positive previous connections often allows for smoother future relationships. In line with the literature on knowledge networks, geographical proximity shows a positive effect on co-authorship prediction, confirming that partnerships between universities in co-authored publications are fostered by proximity. It is interesting to note how the core funding, the size and the reputation play an important but secondary role in predicting collaborations. Finally, the features that measure the environment within which the university is located (GDP and population density) have less predictive importance.

### The predictability of the links and the feature-importance in physical and engineering sciences (PE)

**Figure 3:**
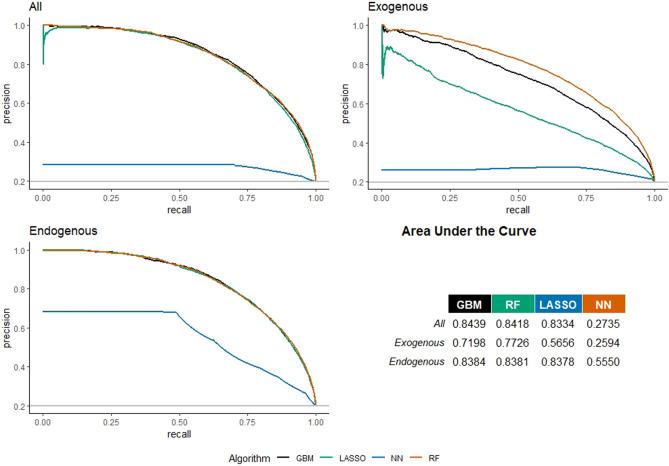
Precision–recall curves for European universities’ co-publication prediction in the Physical and Engineering Sciences. Three models considered: with all, with only exogenous, and with only endogenous features.

Figure 3 shows the Precision-Recall curves for the link prediction in Physical and Engineering Sciences. With regard to the model with all features, the best algorithm in terms of AUC is the GBM (score equal to 0.8439), while considering only exogenous features, the best model in terms of AUC is the RF (score equal to 0.7726); finally, when we consider only the endogenous features, the best model is the GBM with an AUC of 0.838. Also in this case, AUCs are lower than the AUC of the best ML algorithm (GBM) with existing classification methods: AUC for Logistic regression = 0.8376; AUC for Naive Bayes regression = 0.8071. When the endogenous features are removed, the AUC drops down by 8%, while when retaining only network information the worsening is smaller (1%). This result shows that network-related features matter more than the other features to predict co-authorship in PE.

For the three models, as illustrated for the SSH sector, Table 4 lists a set of models’ accuracy performance statistics for the best selected algorithms. As before, all of them confirm that the model including all features outperforms the model with only endogenous features, which in turn outperforms the model with exogenous features. This is an additional proof of the relevance of network information for link prediction. Differently from SSH area (Table 3), in the case of Physical and Engineering Sciences the accuracy of link prediction is higher than the no-information rate for all models (all, only exogenous, only endogenous features). Therefore, co-authorship prediction in the case of PE is more accurate than that obtained for the SSH scientific domain. The latter domain is characterized by a greater heterogeneity in the collaboration behaviours (e.g. different publishing practices, smaller research groups, focus on national topics, etc.), and the considered predictors have a poorer ability to explain co-authorship likelihood, thereby requiring more/different information to reach sizeable accuracy in this scientific domain.

**Table 4:** Accuracy performance indicators for European universities’ co-publication prediction in Physical and Engineering Sciences.

Model	All	Exogenous	Endogenous
Best algorithm	GBM	RF	GBM
ROC	0.9416	0.9173	0.9370
Accuracy	0.8668	0.8406	0.8691
95% CI	(0.8632, 0.8703)	(0.8368, 0.8444)	(0.8655, 0.8726)
No information rate	0.8012	0.7978	0.8007
P-value [Acc > NIR]	0.0000	0.0000	0.0000
Sensitivity	0.8695	0.8418	0.8745
Specificity	0.8562	0.8361	0.8474
Pos pred value	0.9606	0.9530	0.9584
Neg pred value	0.6194	0.5725	0.6270
Prevalence	0.8012	0.7978	0.8007
Detection rate	0.6966	0.6716	0.7002
Detection prevalence	0.7252	0.7047	0.7306
Balanced accuracy	0.8628	0.8389	0.8609

Three models considered: with all, with only exogenous, and with only endogenous features.

**Figure 4:**
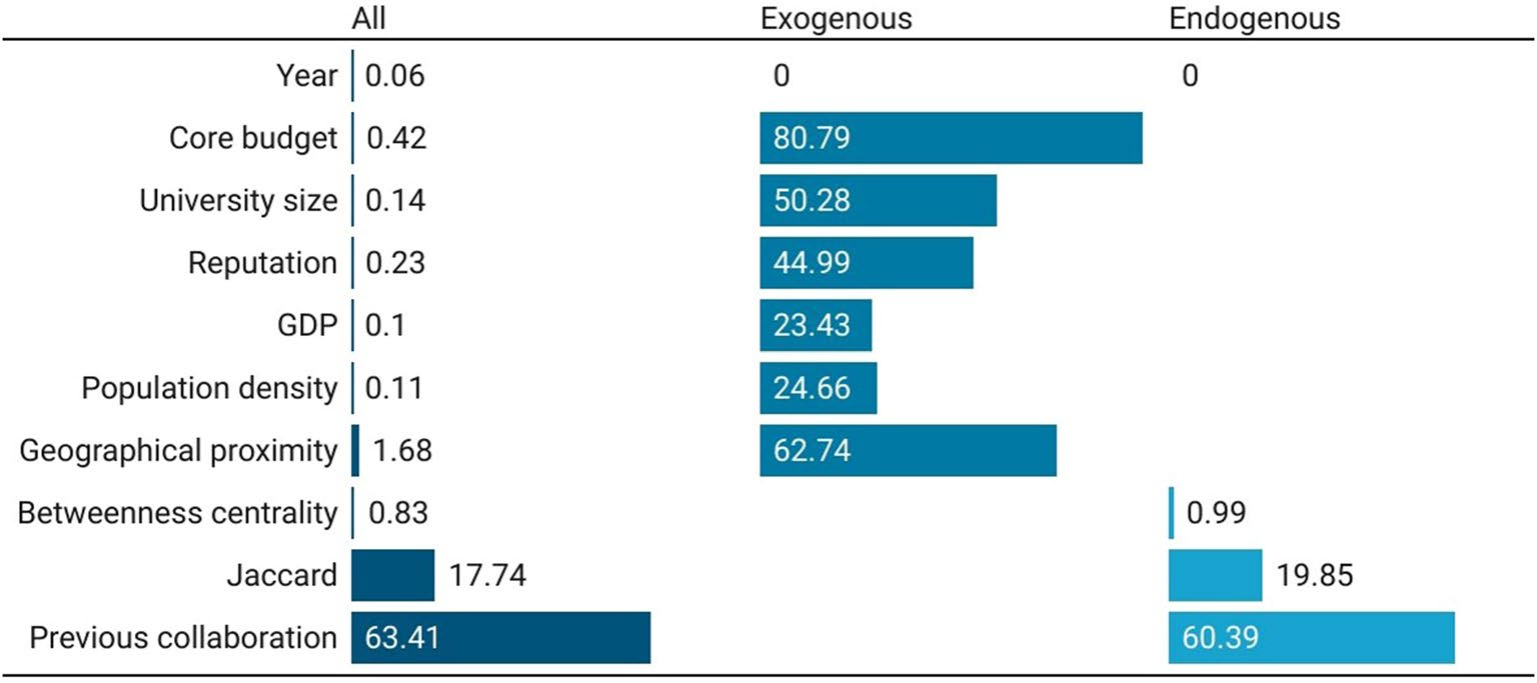
Feature-importance scores for European universities’ co-publication prediction in Physical and Engineering Sciences. Three models considered: with all, with only exogenous, and with only endogenous features. For convenience, features’ importance is averaged across years and universities to be better presented graphically.

Best algorithm’s feature importance for Physical and Engineering Sciences are set out in Fig. 4. The model including all features shows that the previous collaboration is the most important feature (score equal to 63.41), followed by the Jaccard coefficient (17.74), and geographical proximity (1.68). This is an additional confirmation that network matters more than other features in predicting co-authorship. Analyzing only exogenous, the core budget becomes the most important feature to predict links, followed by the geographical proximity, the size, and the reputation of the university. In the PE area, the core funding is markedly more relevant to predict connections when compared to SSH. A possible explanation for this evidence may be found in the higher funding needed for costly equipment and research facilities (e.g. infrastructures, laboratories) that distinguish this scientific domain. Of course, previous collaboration (60.39) and the Jaccard coefficient (19.85) become the most important feature when exogenous features are not included in the model. Unlike SSH, in PE there is greater continuity in collaborations. Moreover, the addition of endogenous factors changes the weight of core funding in terms of prediction, indicating a dependency or spurious effect.

### The predictability of the links and the feature-importance in life sciences (LS)

**Figure 5:**
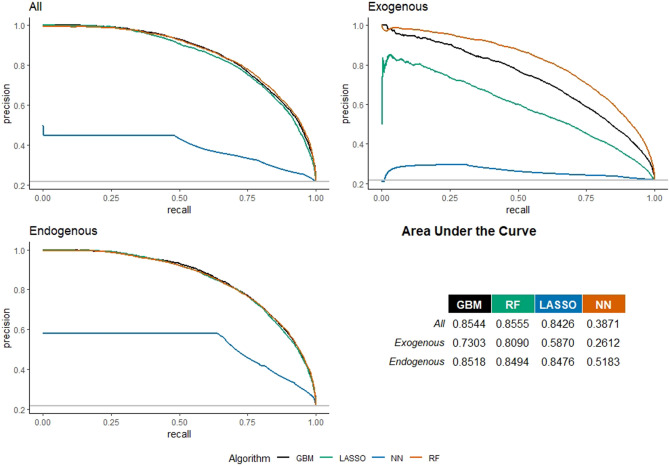
Precision–recall curves for European universities’ co-publication prediction in the Life Sciences. Three models considered: with all, with only exogenous, and with only endogenous features.

Figure 5 shows the Precision–Recall curves for co-authorship prediction in Life Sciences. Using all features, the best algorithm in terms of AUC is the RF (with score equal to 0.8555); by considering only exogenous features, the best model in terms of AUC is the RF (0.8090); finally, considering only endogenous features, the best model is the GBM with an AUC equal to 0.8518.As before, AUCs are lower than the AUC of the best ML algorithm (RF) with existing classification methods: AUC for Logistic regression = 0.8425; AUC for Naive Bayes regression = 0.8189. When the endogenous features are removed, the decrease in the AUC is around 5%, while by retaining only endogenous features this decrease is smaller than 1%. This confirms that, in Life Sciences, the bulk of information needed for predicting co-authorship comes mainly from network characteristics.

For Life Sciences, Table 5 illustrates models’ accuracy performances in predicting co-authorship. All the measures presented in Table 5 confirm that the model with all features outperforms the model with only endogenous features, which in turn outperforms the model with only exogenous features. Furthermore, the accuracy of the best algorithm for all models in Table 5 is statistically higher than the no-information rate. This means that, unlike the SSH but similarly to the PE, link prediction in the Life Sciences is improved by the additional information brought by the features (either exogenous or endogenous) we have consider as co-authorship predictors.

**Table 5:** Accuracy performance indicators for European universities’ co-publication prediction in Life Sciences.

Model	All	Exogenous	Endogenous
Best algorithm	GBM	RF	GBM
ROC	0.9436	0.9257	0.9403
Accuracy	0.8673	0.8464	0.8695
95% CI	(0.8637, 0.8709)	(0.8425, 0.8502)	(0.8659, 0.8731)
No information rate	0.7817	0.7821	0.7836
P-value [Acc > NIR]	0.0000	0.0000	0.0000
Sensitivity	0.8691	0.8472	0.8749
Specificity	0.8612	0.8436	0.8502
Pos pred value	0.9573	0.9511	0.9548
Neg pred value	0.6475	0.6059	0.6524
Prevalence	0.7817	0.7821	0.7836
Detection rate	0.6793	0.6626	0.6855
Detection prevalence	0.7097	0.6967	0.7179
Balanced accuracy	0.8651	0.8454	0.8625

Three models considered: with all, with only exogenous, and with only endogenous features.

**Figure 6:**
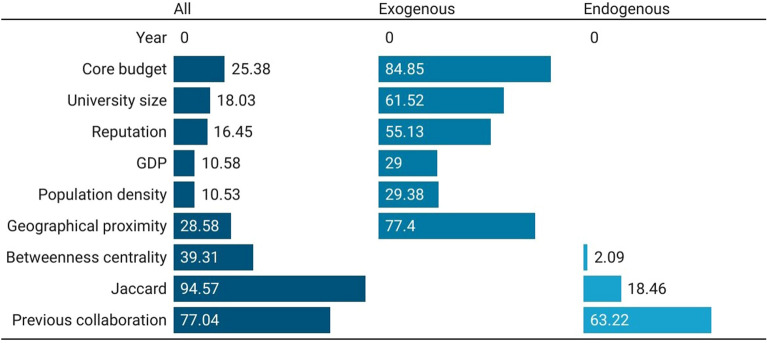
Feature-importance scores for European universities’ co-publication prediction in Life Sciences. Three models considered: with all, with only exogenous, and with only endogenous features. For convenience, features’ importance is averaged across years and universities to be better presented graphically.

Figure 6 shows the feature-importance score for LS in the best algorithm used in the three models (again, with all features, only exogenous features, and only endogenous features). The model with all features show that the Jaccard coefficient is the most important feature (94.57), followed by previous collaboration (77.04), and betweenness centrality (39.31). Also in this case, the three most important features are related to network information, meaning that network matters more than other features for co-authorship prediction. Similarly to the PE domain, among the exogenous attributes, the core budget is the most important feature, followed by the geographical proximity, the size and the reputation of the university. In the endogenous predictors model, the most important feature is the previous collaboration (63.22). It is easy to see that, compared to the model including all the features, the Jaccard coefficient becomes in this case less significant for prediction, in favour of the presence of previous collaborations. This scientific domain is characterized by a large number and more sizable projects from which co-publications derive, running for many years, and establishing collaborations with well-consolidated groups of partners.

In order to explain the differences that emerged between the PE and the LS domains compared to the SSH domain, we can refer to the major complexity of the types of organizations and research practices taking place within PE and LS, compared to SSH. These consist of collaborations that often operate through large R &D laboratories carrying out more explorative and risky research, characterized by larger irreversibility and huger sunk costs. These elements tend to give collaboration patterns a more steady nature, which translates into better predicting performance.

### The predictability of the links and the feature-importance in multidisciplinary science

**Figure 7:**
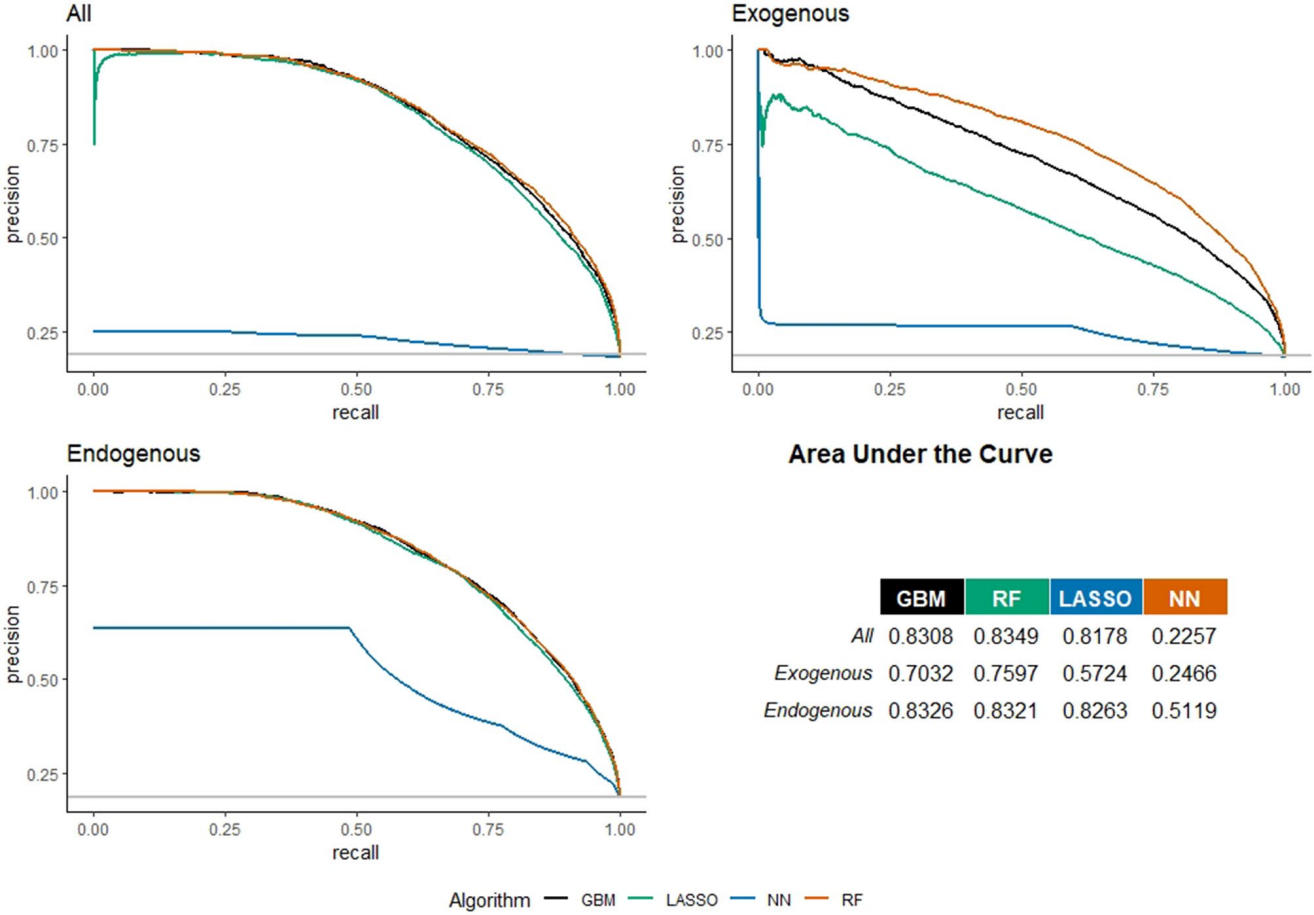
Precision–recall curves for European universities’ co-publication prediction in Multidisciplinary Science. Three models considered: with all, with only exogenous, and with only endogenous features.

Figure 7 shows the Precision-Recall curves for the three models (with all, only with exogenous, and only with endogenous features) in the case of the link prediction in the Multidisciplinary Science. Regarding the model with all features, the best algorithm in terms of AUC is the RF (0.8349); considering only the exogenous features, the best model in terms of AUC is the RF (0.7597); finally, considering only the endogenous features, the best model is the GBM with an AUC of 0.8326.As in the previous three cases, AUC are lower than AUC of the best ML algorithm (RF) with existing classification methods: AUC for Logistic regression = 0.8187; AUC for Naive Bayes regression = 0.7975. There is a sizable worsening in AUC when the endogenous features are removed (9%), while by excluding exogenous (that is, retaining only network information), the AUC is in line with the AUC of the model with all features. This confirms that the large part of information needed for link prediction in the multidisciplinary sector comes from the network. Indeed, the model only using network information sets out a performance which, in terms of AUC, is the same of the model with all features.

Table 6 shows the three models’ performances of the best algorithm used in the link prediction for the Multidisciplinary Science sector. All the measures presented in Table 6 confirm that the model with all features outperforms the model with only endogenous features, which in turn outperforms the model with exogenous features. Overall, the accuracy is higher than the no-information rate for all models (all, only exogenous, only endogenous). This means that, in line with the link prediction for PE and LS, the link prediction in Multidisciplinary Science is significantly accurate.

**Table 6:** Accuracy performance indicators for European universities’ co-publication prediction in Multidisciplinary Science.

Model	All	Exogenous	Endogenous
Best algorithm	GBM	RF	GBM
ROC	0.9418	0.9227	0.9357
Accuracy	0.8654	0.8354	0.8644
95% CI	(0.8615, 0.8692)	(0.8312, 0.8396)	(0.8605, 0.8682)
No information rate	0.8171	0.8158	0.8120
P-value [Acc > NIR]	0.0000	0.0000	0.0000
Sensitivity	0.8659	0.8308	0.8687
Specificity	0.8633	0.8560	0.8457
Pos pred value	0.9659	0.9623	0.9605
Neg pred value	0.5903	0.5332	0.5986
Prevalence	0.8171	0.8158	0.8120
Detection rate	0.7075	0.6778	0.7053
Detection prevalence	0.7325	0.7043	0.7344
Balanced accuracy	0.8646	0.8434	0.8572

Three models considered: with all, with only exogenous, and with only endogenous features.

**Figure 8:**
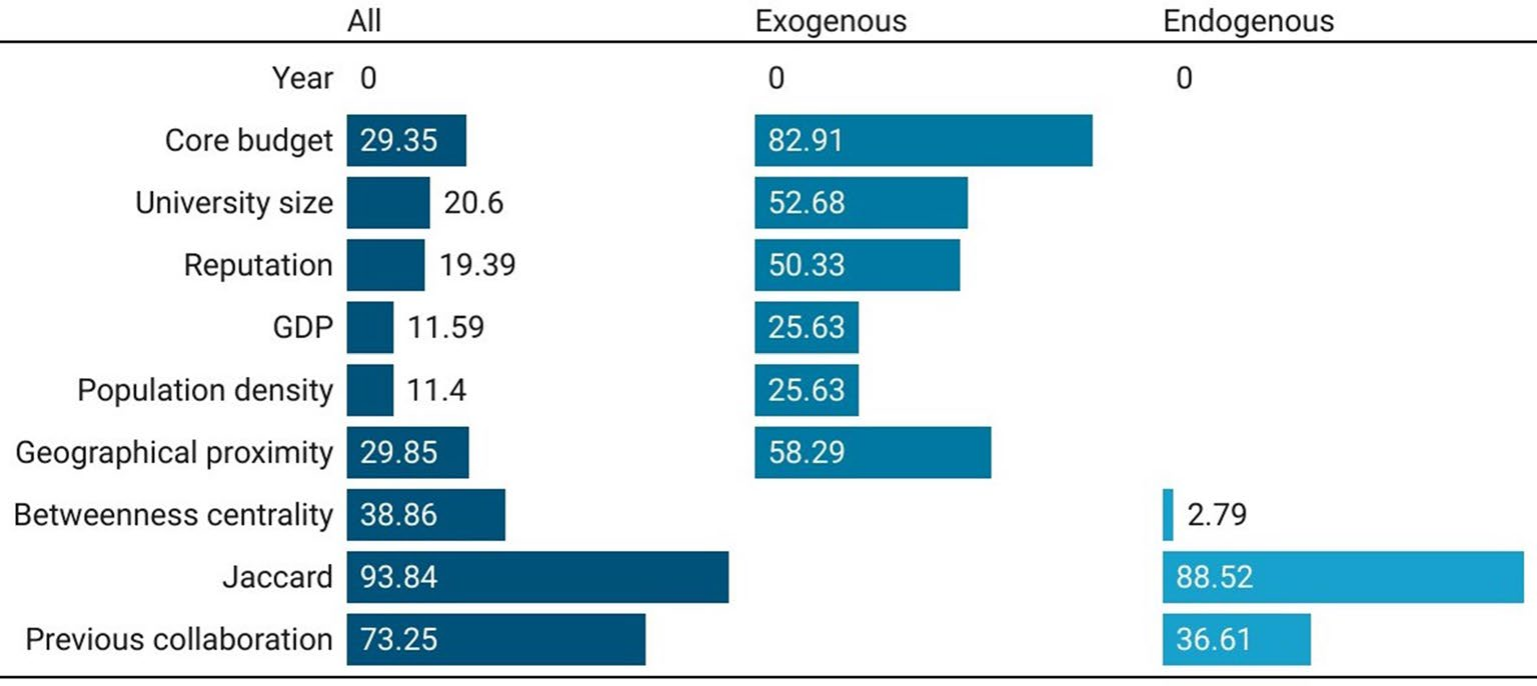
Feature-importance scores for European universities’ co-publication prediction in Multidisciplinary Science. Three models considered: with all, with only exogenous, and with only endogenous features. For convenience, features’ importance is averaged across years and universities to be better presented graphically.

Figure 8 shows feature-importance scores for co-authorship prediction in the Multidisciplinary Science for the best algorithm in the three specifications. The model with all features shows that the Jaccard coefficient is the most important feature (93.84), followed by the presence of previous collaborations (73.25), and the betweenness centrality (38.86). It is worth noting that the three most important features are connected, also for this domain, to network information. When we exclude from the specification the network features, we see that the core budget becomes the most important feature to predict co-authorship, followed by the distance, the number of students, and the reputation. The Jaccard coefficient (88.52) becomes significantly more predictive of the previous collaboration (36.61) when exogenous features are not included in the model.

## Discussion

Scientific collaboration is becoming more common and important. As a result, there is a growing interest in learning more about its causal factors. In this paper, we estimated link occurrence and computed prediction feature-importance scores in four university co-publication networks: Social Sciences and Humanities (SSH), Physical and Engineering Sciences (PE), Life Sciences (LS) and Multidisciplinary Sciences (MS).

We found a sizable co-publication prediction accuracy, larger than 80%, over all ERC domains and specifications. Our initial hypothesis regarding government funding was verified. We observed that compared to SSH, government funding is more significant in PE and LS. Additionally, our third hypothesis-that the investigated knowledge networks’ structural characteristics have a greater impact on predictive performance than exogenous factors—is confirmed.

Both in terms of accuracy and features’ importance, we observed differences among domains, due to the different knowledge production modes taking place within them. The presence of large laboratories and research infrastructures characterizing the PE and LS domains, as well as the nature of the knowledge generated in these domains (typically more codified, storable, and transferable) compared to the SSH domain may certainly explain some of these differences. Researchers working in the PE and LS domains typically operate through more stable and lasting partnerships in collaborative join works. In SSH, in contrast, there is a weaker tendency of scholars to collaborate in large groups and for a long time, and academics in this field prefer different types of publication outputs, such as books or conference proceedings.

These different scientific communities are also characterized by diversified intellectual and organizational patterns, based on a different degree of mutual dependence and uncertainty of the scientific outcomes^67^.

The SSH domain differs from the other domains as the geographical proximity has a stronger impact on predicting collaborations. In contrast, co-authorship prediction in the PE domain shows a higher intertemporal dependence: the presence of past collaborations has greater impact on future co-authorship. In the LS domain, for the general model, the Jaccard coefficient stands out as the most important feature, while in the model with only endogenous features, previous connections become the most relevant attribute. Finally, in the LS and MS co-publication domains, the betweenness centrality has higher predictive power than in SSH and PE: universities belonging to, and in connection with, a larger number of communities tend to collaborate more in multidisciplinary papers.

All our results converge to stress the outstanding role played by the degree of *embeddedness* within the scientific community—measured by the Jaccard coefficient—to predict new connections. Indeed, for explaining the likelihood of co-publication formation, the community structure seems to be more significant than the level of government funding, the regional economic wealth, and the geographical proximity.

Our results raise a general concern that goes beyond co-publication prediction. Indeed, investigating the determinants of prospective scientific collaborations is an issue of policy relevance, necessary for suitably designing future science policies that aim at improving competitive project funding and sustaining academic research and universities’ performance.

## Conclusions

Our results help to examine the value of the network and non-network variables in the formation of scientific collaborations in Europe. We have seen that some variables—both endogenous and exogenous—appear to be important in all scientific disciplines under consideration: the Jaccard coefficient, the presence of past collaboration, and the core funding. Among all the examined factors, the Jaccard coefficient appears to be the most relevant. It ranks second only for the PE domain. The Jaccard coefficient can be considered a proxy of an epistemic group membership. The main result is that the scientific community could be helpful in finding productive collaborations, which would foster the formation of strong research teams. Our findings also imply that these groups may play a long-term role in promoting collaborations. This can be concluded from the observation that past collaboration increases the likelihood of future collaboration. These two results suggest that the community structure has been mainly preserved over time. As a result, the four networks expand in a self-similar way. These findings also highlight a number of challenges, including the need to redefine or rethink some of the policy implementation strategies to favor the inclusion of those researchers and universities that are less embedded in research initiatives. In addition, we focused on the role of funding in all the analyzed scientific fields. We find that academics who work in universities and receive more government funding are also more likely to collaborate. Moreover, funding makes it possible for researchers to work together more frequently by removing the barriers posed by geographical distance. In fact, funding can enable attendance at conferences and workshops, which encourages in-person interactions, thus promoting the setup of collaborations. Government funding also makes research infrastructure and communal areas (like labs) accessible, encouraging collaboration. This should be considered when developing research policies that should aim at favoring the most disadvantaged universities by mitigating the Matthew effect of accumulated advantage. Universities that have previously been successful in scientific collaborations are more likely to do it again, thereby producing an increasing distinction. This is because a quota of the institutional funding system is performance-based, making the Matthew effect drive the allocation of research funds. The assumption that government funding will benefit universities evenly and uniformly is no longer appropriate. Future R &D policies should take into account these potential differences. Taking into account the role played by network dynamics in the different scientific fields can thus help to implement policy strategies to overcome the “rich club” or “Matthew effect” phenomena, as well as to favor the transmission of tacit knowledge and experience sharing also towards the most peripheral nodes of the network.

## Data Availability

Original data sources are from the European Tertiary Education Register project (https://eter-project.com/). These are fully public data. Bibliometric data are from the CWTS, University of Leiden and can be requested through the EU-FP RISIS2 project (https://rcf.risis2.eu/datasets) for research purposes. The authors did not have special access privileges to access to these data. These data allow for reproducing the whole research and, in particular, generating all variables for the study.
